# Parents’ Uptake and Willingness towards Recommended Vaccinations for Their Children with Underlying Chronic Medical Conditions in Italy

**DOI:** 10.3390/vaccines11091423

**Published:** 2023-08-27

**Authors:** Giorgia Della Polla, Grazia Miraglia del Giudice, Mario Postiglione, Italo Francesco Angelillo

**Affiliations:** 1 Department of Public Health and Laboratory Services, Teaching Hospital of the University of Campania “Luigi Vanvitelli”, Via Luciano Armanni 5, 80138 Naples, Italy; 2 Department of Experimental Medicine, University of Campania “Luigi Vanvitelli”, Via Luciano Armanni 5, 80138 Naples, Italy

**Keywords:** children, chronic condition, Italy, uptake, vaccinations, willingness

## Abstract

This cross-sectional survey was conducted to investigate the willingness and uptake of recommended vaccinations against influenza, meningococcal B and ACWY, pneumococcal, rotavirus and the influencing factors among 565 parents of children aged 6 months to 5 years with chronic medical conditions in Italy. Only 34.9% of the sample received all vaccinations. Parents whose selected child was vaccinated against the five diseases were those who had received recommendations from physicians, who did not believe that children should get fewer vaccinations at the same time, those whose child was aged 2–3 and 4–5 years compared to 6 months–1 year, and those who acquired information from physicians. Only 17.9% were willing to vaccinate their child. Parents with a university degree, those who acquired information from physicians, and those whose child had a more recent diagnosis were more likely to be willing to vaccinate their child. Parents who believed that children should get fewer vaccines at the same time, those without a university degree, and those who did not acquire information from physicians were more likely to not have vaccinated their child because they were concerned about vaccines’ side effects. Public health policymakers should provide efforts to promote the uptake for an adequate protection of this high-risk group.

## 1. Introduction

Over the past decades, the prevalence of children with chronic medical conditions has progressively increased with the risk of experiencing complications including more severe and long-term illness, hospitalization, and even death [1,2,3]. Moreover, it is well known that the introduction of vaccines, coupled with the implementation of programs aimed at universal infant immunization, have reduced the incidence of several vaccine-preventable diseases and their related complications [4].

In Italy, according to the National Immunization Prevention Plan, ten mandatory vaccinations against diphtheria, *Haemophilus influenzae* serotype b, hepatitis B, pertussis, polio, tetanus, chickenpox, measles, mumps, and rubella are provided free of charge for all children and four are recommended against meningococcal, pneumococcal diseases, rotavirus, and seasonal influenza for children with underlying chronic medical conditions [5]. However, despite these recommendations, pneumococcal, meningococcal B and ACWY vaccination uptake is suboptimal among children aged 48 months, still lower than the required rate of 95%, with the values of 91%, 73.7%, and 48.6%, respectively [6]. This data is alarming because even lower coverage rates have been observed among children with chronic medical conditions compared to those who are healthy [7,8,9]. This makes vaccine uptake a crucial public health priority for children with chronic medical conditions.

It is well known that the intentions of parents toward vaccinations and their related reasons are extremely relevant for achieving higher coverage rates. Previous investigations have focused their attention on the attitudes and behaviors of parents in relation to different vaccinations for their children with chronic medical conditions [7,8,10,11], but to the best of our knowledge only few surveys have examined this topic in Italy [12,13]. Undoubtedly, this information is extremely important for the development of vaccination campaigns for fostering acceptance. Given the lack of literature, the aims of this cross-sectional survey were to investigate the willingness and uptake of the recommended vaccinations and the factors that facilitate or hinder these vaccinations among parents of children with chronic medical conditions in Italy.

## 2. Materials and Methods

### 2.1. Setting and Sample

Data were collected as part of a larger survey that examined perceptions and behaviors of parents towards the recommended vaccinations for their children [12,14,15,16]. The survey was conducted from February to April 2023 in a teaching and in a public hospital randomly selected in the city of Naples, Southern part of Italy. All parents with a child aged between 6 months to 5 years with a chronic medical condition who attended pediatric outpatient clinics were enrolled. A childhood chronic condition is defined as a disease that can be diagnosed according to professional standards with an expected duration of at least 3 months or the impossibility of a cure [17].

The minimum required sample size was estimated to be 427, on the assumption that 50% of the parents had vaccinated their children for all recommended vaccines, with a confidence interval of 95%, a margin of error of 5%, and an expected response rate of 90%.

### 2.2. Data Collection

The protocol and questionnaire were approved prior to survey commencement by the Ethics Committee of the Teaching Hospital of the University of Campania “Luigi Vanvitelli” (protocol number 0001816/i). Prior to the start of the investigation, the research team sent a letter of invitation to the health directors of the selected hospitals which asked permission to carry out the survey and explained purposes and procedures. Once the authorization was obtained, each potential participant was approached while waiting for their child’s appointment at the outpatient clinic by trained investigators for a face-to-face interview or contacted by telephone from Monday to Friday between 10:00 a.m. and 7:00 p.m., ensuring the inclusion and participation of working and non-working parents. All potential participants were informed about the survey’s design and purposes, that the participation was voluntary, that anonymity and confidentiality of the data were guaranteed, that information would be used for scientific purposes, and they were assured that they could terminate the participation at any time without justification. In the case of more than one child with a chronic medical condition, participants were requested to respond about their child closer to six months of age. Verbal informed consent was obtained from participants before the interview. All parents were not compensated for their participation.

### 2.3. Questionnaire

The questionnaire was modified and adapted from those used by some of us in previous surveys aimed at evaluating parental perception and behaviors regarding the vaccinations for their children [12,14,15,16]. A total of 10 participants participated in the pilot phase to evaluate the questionnaire’s consistency and wording. Since no changes were made to the questionnaire items, the results were included in the analysis. The questionnaire was structured in three parts and required approximately 10 min to be answered. The first part collected the socio-demographic and health-related characteristics of the respondents (i.e., age, gender, marital status, educational level, occupation, history of chronic medical conditions) and of the selected children (i.e., age, gender, chronic medical conditions). The second part collected the attitudes regarding the recommended vaccinations for the selected child measured on a five-point Likert scale ranging from 1 = strongly disagree to 5 = strongly agree. Parents were also asked to report which recommended vaccinations of the five (against rotavirus, seasonal influenza, meningococcal, and pneumococcal diseases) the child had received, and the response options were “yes”, “no”, and “do not remember”. Parents who had not vaccinated their selected child were asked about their intentions to vaccinate the child, with “yes”, “no”, and “do not know” responses, against seasonal influenza, meningococcal, and pneumococcal diseases and not against rotavirus since it is recommended until 6 months of age. They were asked to select the reasons for their willingness for or unwillingness against the different diseases from a list of 8 reasons why they might want or not want their children to receive each vaccine. The third part asked which sources participants used for acquiring information about the recommended vaccinations for the child and whether they needed additional information.

### 2.4. Statistical Analysis

Statistical analyses were conducted using the STATA software version 17(College Station, TX, USA) [18]. First, frequency, mean, range, and standard deviation were used to describe the sample characteristics, attitudes, and behaviors. Second, univariate analysis using chi-square test or Student’s *t*-test was performed to assess the associations between categorical and continuous variables and the outcomes of interest, respectively. Third, the independent variables with a *p*-value ≤ 0.25 in the univariate analysis were entered in three multivariate logistic regression models to evaluate the variables that were independently significantly associated with the following outcomes of interest: parents who had vaccinated their selected child with all recommended vaccinations (Model 1); parents’ willingness to vaccinate their selected child with the recommended vaccines not already received (against seasonal influenza, meningococcal, and pneumococcal diseases) (Model 2); and parents who do not intend to vaccinate their selected child because of concerns about vaccine’s side effects (Model 3). The following independent variables regarding the respondent parent have been tested for all outcomes: gender (male = 0; female = 1), age in years (continuous), marital status (unmarried/separated/divorced/widowed = 0; married/cohabited with a partner = 1), having a baccalaureate/graduate degree (no = 0; yes = 1), employment status (unemployed = 0; employed = 1; healthcare worker = 2), having at least one chronic medical condition (no = 0; yes = 1), believing that children get more shots than are good for them (strongly disagree/disagree/uncertain = 0; agree/strongly agree = 1), believing that it is better for their child to develop immunity by getting sick than to get a shot (strongly disagree/disagree/uncertain = 0; agree/strongly agree = 1), believing that children should get fewer vaccines at the same time (strongly disagree/disagree/uncertain = 0; agree/strongly agree = 1), having received recommendations to immunize their child for all vaccines (no = 0; yes = 1), having acquired information about the recommended vaccines for their child from physicians (no = 0; yes = 1), and need of additional information (no = 0; yes = 1). The following are the independent variables regarding the selected child: age (6 months-1 year = 0; 2–3 years = 1; 3–5 years = 2), gender (male = 0; female = 1), only child (no = 0; yes = 1), firstborn (no = 0; yes = 1), having a chronic medical condition that causes immunodeficiency (no = 0; yes = 1), length of time from the diagnosis of the chronic medical condition, in months (continuous), current use of medication(s) (no = 0; yes = 1), having more than one chronic medical condition (no = 0; yes = 1), and number of pediatric visits in the last year (continuous). The values of *p* = 0.2 and *p* = 0.4 were used to select candidate variables for retention and exclusion in the final multivariate logistic regression models. Results of the logistic regression models were measured using odds ratios (OR) with 95% confidence intervals (CI). For all analyses, two-tailed tests were used and *p*-values ≤ 0.05 were considered statistically significant.

## 3. Results

A total of 565 questionnaires were returned, representing 95% of the 595 parents approached. The main characteristics of the respondent parents and of the selected child are given in Table 1. Most parents were female and married or living with a partner, the mean age was 36 years, less than one-third had at least a university degree, less than half were employed, and 8.3% had at least one chronic medical condition. The selected children were nearly equally distributed by sex, the mean age was 2.6 years, the most frequent causes of frailty were kidney diseases (27.3%), prematurity (23.2%), and cardiovascular diseases (18.2%), and 3% had more than one chronic medical condition.

For survey items on parents’ attitudes towards the recommended vaccinations, 13.3% agreed that children receive more recommended vaccines than are good for them, 10.4% thought that it was better for their child to develop immunity by getting sick than by getting a shot, 29.8% believed that children should get fewer vaccines at the same time, 10.1% had refused at least one vaccination for their child. The majority confirmedthat they received recommendation from a physician for at least one vaccine (83.2%), mainly against meningococcal B (81.4%), followed by meningococcal ACWY (72.8%), influenza (52.8%), pneumococcal (45.6%), and rotavirus (43.5%). However, for only one-fourth (24.5%) the recommendation was for all vaccines. The highest self-reported coverage was for the meningococcal B (74.5%), followed by pneumococcal (62.8%), meningococcal ACWY (62.1%), rotavirus (57.5%), and influenza vaccine (45.8%). Overall, only 34.9% stated that their child had received all five vaccines. Three multivariate logistic regression models were fitted to assess which factors were more likely to affect the outcomes of interest and the results are displayed in Table 2. Parents whose selected child was vaccinated against the five diseases were those who had received the recommendation by a physician to immunize their child for all vaccines (OR = 15.75; 95% CI = 8.82–28.15), those who did not believe that children should get fewer vaccines at the same time (OR = 0.21; 95% CI = 0.11–0.41), and those whose child was aged 2–3 years (OR = 2.34; 95% CI = 1.27–4.31) and 4–5 years (OR = 3.74; 95% CI = 2.02–6.93) compared to those whose child was aged 6 months-1 year. Parents who had acquired information about the recommended vaccines for their child from physicians were 5.5 times (95% CI = 1.72–17.72) more likely to have vaccinated their child with all those recommended compared to those who did not utilize this source (Model 1). 

Among those parents whose selected child was not vaccinated against at least one disease, only 17.9% indicated that they were willing to vaccinate their child. In the multivariate logistic regression analysis, four factors were significantly associated with parents’ willingness to vaccinate their child. The respondents with a university degree (OR = 1.95; 95% CI = 1.04–3.66), those who had acquired information about the recommended vaccines for their child from physicians (OR = 3.77; 95% CI = 1.09–12.97), those who had received the recommendation from a physician to immunize their child for all vaccines (OR = 2.52; 95% CI = 1.08–5.91), and those who had a child with a more recent diagnosis of a chronic medical condition (OR = 0.96; 95% CI = 0.93–0.98) were more likely to be willing to vaccinate their child (Model 2 in Table 2). The most common reason reported by parents for their willingness to vaccinate their child was to protect them (74.2%), followed by receiving a recommendation from pediatricians (31.8%), whereas the most frequent reasons for not getting the vaccinations were believing that their child was not at risk (28.5%) and concerns about the vaccines’ side effects (22.2%). Parents who believed that children should get fewer vaccines at the same time (OR = 2.11; 95% CI = 1.14–3.91), those who did not have a baccalaureate/graduate degree (OR = 0.26; 95% CI = 0.11–0.61), and those who did not acquire information about the recommended vaccines for their child from physicians (OR = 0.42; 95% CI = 0.21–0.85) were more likely to have not vaccinated their child because they were concerned about vaccines’ side effects (Model 3 in Table 2).

Most of the sample had gained information about the recommended vaccinations for their child (90.4%) and almost all of them had used the pediatricians as a source (93.9%). Finally, one-third (31.6%) expressed the need for more information on this topic.

## 4. Discussion

The current cross-sectional survey is the most comprehensive among parents in Italy regarding attitudes and behaviors and further the potentially influential factors of vaccination for their children with chronic medical conditions. To the best of our knowledge, this is the first survey of its kind to be completed in Italy and, therefore, it is presently unclear how much information is appreciated among the parents and whether there is willingness to vaccinate their children. A better understanding of this topic is crucial for controlling the vaccine-preventable diseases.

The first key finding was that only 34.9% of the parents reported that their child with chronic medical conditions had received all recommended vaccinations and the coverage ranged from 45.9% for influenza to 74.5% for meningococcal B. The observed coverages were higher than those found in studies conducted in Italy among parents of healthy children in which the prevalence was 29.9% for influenza [19] and 15.3% for rotavirus [20]. It is worrying to observe that for none of the recommended vaccines has the target coverage rates indicated in the National Immunization Prevention Plan been achieved [5], although most of the sample had gained information about these vaccines for their child (90.4%) and almost all of them had used the pediatricians as source (93.9%). Healthcare workers have an important role since the results of the multivariate logistic regression analysis showed that having received a recommendation to immunize their child from physicians was one of the crucial factors influencing the parents’ behaviors and the positive intention to receive the recommended vaccinations. Indeed, parents who had received a recommendation and those who had acquired information from physicians were more likely to have vaccinated their child and to intend to vaccinate their child with all recommended vaccinations. These findings highlight the central role played by physicians in the parents’ education and decisions and confirm previous studies that showed the positive influence of receiving a physicians’ recommendation on the populations’ attitudes and behaviors with a higher vaccination willingness and coverage [15,19,21,22,23,24]. However, it is necessary to underline that only one-fourth of the sample had received a recommendation from physicians and, therefore, it is imperative to provide educational activities to the healthcare workers particularly aiming to raise their awareness about vaccines’ efficacy and to recommend vaccinations for children with chronic medical conditions. Furthermore, parents who did not acquire information from physicians were more likely to have not vaccinated their child for the concerns about the side effects. Finally, in this survey a higher vaccination rates were also observed among parents with a child aged 2–3 and 4–5 years compared to those whose child was aged 6 months-1 year. The finding that parents with an older child were more likely to have immunized him/her has already been reported in other studies [25,26,27]. This is not surprising because, as might be expected, older children have already received all ten mandatory vaccines included in the routine immunization schedule, hence parents could be more inclined to also vaccine their younger child for the recommended ones.

The second key finding was that only 17.9% of the parents with under-vaccinated children responded that they would vaccinate their child with all recommended vaccines. Of the several sociodemographic characteristics, only the parents’ level of education was significantly associated with this outcome of interest. Indeed, having a university-level education was related to having a higher willingness to vaccinate their child. This is in line with the results of other surveys [26,28,29,30,31]. The positive attitude in the highly educated parents may be because they were well informed and educated with a higher level of understanding of the benefit of the vaccines for their child’s health conditions and, therefore, they are able to make a more informed choice. Moreover, it is worth noting that parents having a child with a more recent diagnosis of the chronic medical condition were more likely to intend to immunize him/her. It is reasonable to assume that this parents’ favorable willingness could be explained by their fear for the “new” condition of the children and by their perception of disease threats. In this survey, the primary reason for their willingness to accept the vaccinations was to safeguard the child, whereas the major reasons for rejecting immunization were that their child was not at risk and the fear of adverse effects. These results are in line with previous literature on different groups of individuals [14,32,33,34,35]. The underestimated perception of the risk for children with chronic medical conditions requires specific attention and highlights the urge initiatives to be taken and the favorable safety profile of the vaccines need to be shared to make appropriate decisions.

The third key finding was that the multivariate logistic regression analysis shows the importance of various additional respondents’ personal and general characteristics that play a key role in the behaviors and willingness towards the recommended vaccination decisions. Important factors include respondents’ attitudes about vaccinations. Parents who did not believe that children receive more vaccinations that are good for them were more likely to have vaccinated their child for all those recommended, while those who believed that children should get fewer vaccines at the same time were more likely to not intend to vaccinate their selected child because of concerns about vaccines’ side effects. These findings were in alignment with earlier surveys which found that these concerns were the main reasons for not accepting vaccination [36,37]. Therefore, as already indicated, public health intervention programs are needed and should be focused on reducing the unjustified perception of the concerns regarding the vaccination and increasing the perception of their benefits. In addition, effective communication activities highlighting the importance of the protection of children with chronic medical conditions and of the community are needed.

It is necessary to recognize some potential methodological limitations of this survey when interpreting the results. First, as for all cross-sectional survey design, a cause-and-effect relationship between independent variables and outcomes cannot be established. Second, the survey enrolled participants in a single area and, therefore, the findings may not be generalized to the whole Italian population of parents of children with chronic medical conditions. Third, the survey used self-reported data, and this may have contributed to potential recall or social desirability bias, because participants may have answered the questions based on what they perceived was expected from them. The children’s immunization date was not verified with the medical records, thus, there may be an overestimation of the uptake. However, since the survey was anonymous and confidential this may have reduced such biases. Despite these limitations, the survey provides valuable insights for policymakers to implement vaccination coverages among children with chronic conditions.

## 5. Conclusions

In conclusion, the results of this survey highlight the factors implicated in the low adherence to the recommended vaccinations among children with chronic conditions. The finding that having received recommendation and information from physicians were significant predictors of parents’ willingness, as well as behaviors regarding the vaccinations for their child, suggests the role played by health care workers in advising and informing is essential. Public health policymakers should provide efforts to involve the parents in educational interventions promoting these recommended vaccinations for improving their understanding of the importance of the vaccinations for these children and for promoting the uptake for an adequate protection of this high risk group.

## Figures and Tables

**Table 1:** Main sociodemographic, general, and anamnesis characteristics of the sample.

Characteristics	*N*	%
**Parent**		
Age, years	36 ± 5.9 (20–60) *	
Gender		
Female	493	87.3
Male	72	12.7
Partnership status		
Married/living with a partner	524	92.7
Unmarried	41	7.3
Educational level		
High school degree or less	399	70.7
Baccalaureate/graduate degree	165	29.3
Employment status		
Unemployed	286	50.7
Employed	251	44.5
Employed as healthcare worker	27	4.8
Having at least one chronic medical condition		
No	517	91.7
Yes	47	8.3
**Selected child**		
Age, years	2.6 ± 1.6 ***	
6 months–1 year	170	30.6
2–3	186	33.5
4–5	199	35.9
Gender		
Female	273	49.4
Male	280	50.6
Only child		
No	281	49.7
Yes	284	50.3
Frailty condition **		
Kidney	154	27.3
Prematurity (<2 years of age)	131	23.2
Cardiovascular	103	18.2
Type 1 diabetes	48	8.5
Hematologic/Oncologic	30	5.3
Gastrointestinal	30	5.3
Other	86	15.3
Length of time from diagnosis of the chronic medical condition, months	22.5 ± 14.9 (1–60) *	
Current use of medication(s)		
No	436	77.2
Yes	129	22.8

Number for each item may not add up to the total number of study population due to missing value; * Mean ± Standard deviation (range); ** More than one chronic medical condition was allowed to be indicated; *** Mean ± Standard deviation.

**Table 2:** Results of the multivariate logistic regression analysis showing the predictors of the different outcomes of interest.

Variable	OR	SE	95% CI	*p*
**Model 1.** Parents who had vaccinated their selected child with all recommended vaccinations Log likelihood = −233.11, χ^2^ = 199.1 (8 df), *p* < 0.0001				
Having received recommendations to immunize their child with all vaccines	15.75	4.66	8.82–28.15	<0.001
Not believing that children should get fewer vaccines at the same time	0.21	0.07	0.11–0.41	<0.001
Child age, in years				
6 months–1 year	1.00°			
2–3	2.34	0.72	1.27–4.31	0.006
4–5	3.74	1.18	2.02–6.93	<0.001
Having acquired information from physicians	5.52	3.28	1.72–17.72	0.004
Not believing that it is better for their child to develop immunity by getting sick than to get a shot	0.37	0.21	0.12–1.11	0.077
Not believing that children get more shots than are good for them	0.58	0.29	0.22–1.54	0.277
Male parents	0.72	0.25	0.37–1.42	0.343
**Model 2.** Parents’ willingness to vaccinate their selected child with all vaccines recommended Log likelihood = −139.28, χ^2^ = 44.89 (8 df), *p*<0.0001				
More recent diagnosis of the chronic medical condition	0.96	0.14	0.93–0.98	0.004
Having received recommendations to immunize their child with all vaccines	2.52	1.09	1.08–5.91	0.033
Having acquired information from physicians	3.77	2.37	1.09–12.97	0.035
Having a baccalaureate/graduate degree	1.95	0.63	1.04–3.66	0.037
Not believing that children get more shots than are good for them	0.39	0.19	0.15–1.04	0.061
Higher number of pediatric visits in the last year	1.03	0.02	0.99–1.07	0.081
Child age, in years				
6 months–1 year	1.00 *			
2–3	0.65	0.23	0.33–1.32	0.236
Only child	1.43	0.44	0.78–2.61	0.248
**Model 3.** Parents who do not intend to vaccinate their selected child because of concerns about vaccine’s side effects Log likelihood = −148.15, χ^2^ = 46.31 (9 df), *p* < 0.0001				
Not having a baccalaureate/graduate degree	0.26	0.11	0.11–0.61	0.002
Not having acquired information from physicians	0.42	0.15	0.21–0.85	0.016
Believing that children should get fewer vaccines at the same time	2.11	0.66	1.14–3.91	0.017
Female parents	2.63	1.53	0.84–8.26	0.098
No need of additional information	0.56	0.21	0.27–1.14	0.109
Not having at least one chronic medical condition	0.47	0.23	0.18–1.21	0.119
Believing that it is better for their child to develop immunity by getting sick than to get a shot	1.75	0.69	0.81–3.79	0.158
Unmarried/separated/divorced/widowed	0.55	0.27	0.21–1.44	0.224
Less recent diagnosis of the chronic medical condition	1.01	0.01	0.98–1.03	0.386

* Reference category.

## Data Availability

The anonymous data presented in this study are available on request from the corresponding author.
